# Structural Basis for the Initiation of Glycosaminoglycan Biosynthesis by Human Xylosyltransferase 1

**DOI:** 10.1016/j.str.2018.03.014

**Published:** 2018-06-05

**Authors:** David C. Briggs, Erhard Hohenester

**Affiliations:** 1 Department of Life Sciences, Imperial College London, London SW7 2AZ, UK

**Keywords:** proteoglycan, glycosyltransferase, enzyme catalysis, X-ray crystallography

## Abstract

Proteoglycans (PGs) are essential components of the animal extracellular matrix and are required for cell adhesion, migration, signaling, and immune function. PGs are composed of a core protein and long glycosaminoglycan (GAG) chains, which often specify PG function. GAG biosynthesis is initiated by peptide *O*-xylosyltransferases, which transfer xylose onto selected serine residues in the core proteins. We have determined crystal structures of human xylosyltransferase 1 (XT1) in complex with the sugar donor, UDP-xylose, and various acceptor peptides. The structures reveal unique active-site features that, in conjunction with functional experiments, explain the substrate specificity of XT1. A constriction within the peptide binding cleft requires the acceptor serine to be followed by glycine or alanine. The remainder of the cleft can accommodate a wide variety of sequences, but with a general preference for acidic residues. These findings provide a framework for understanding the selectivity of GAG attachment.

## Highlights

• The first crystal structure of a peptide *O*-xylosyltransferase has been determined
• The sugar donor, UDP-xylose, is bound in a pocket that excludes other sugar types
• The acceptor peptide is bound in an extended conformation by an active-site flap
• A constriction requires the modified serine to be followed by a small amino acid

## Graphical Abstract

**Figure:**
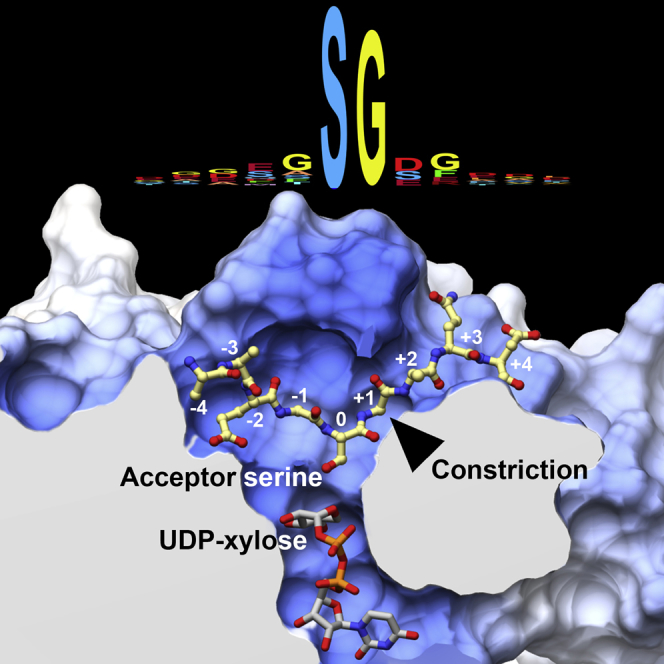

## Teaser

Glycosaminoglycans are sulfated polysaccharides essential for animal life. Their biosynthesis is initiated by peptide *O*-xylosyltransferase, which modifies selected serine residues within proteoglycan core proteins. Briggs and Hohenester have determined crystal structures of human xylosyltransferase 1 that define the catalytic mechanism and substrate specificity of this enzyme.

## Introduction

Proteoglycans (PGs) are a diverse family of glycoproteins characterized by the presence of one or more covalently attached glycosaminoglycan (GAG) chains, which often dominate the biophysical properties and biological functions of PGs. GAGs are polymers of repeating disaccharide units consisting of a hexosamine and a uronic acid sugar; the polymer is modified by sulfation at various positions (Esko et al., 2009, Iozzo and Schaefer, 2015). Depending on the hexosamine, GAGs are classified as either heparan sulfate (HS) or as chondroitin sulfate (CS) and dermatan sulfate (DS). PGs are present on the cell surface and in the extracellular matrix of all higher animals. They are essential components of extracellular matrices and play important roles in cell adhesion and migration, morphogen and growth factor signaling, immune regulation, and the inflammatory response. PG dysfunction is linked to many conditions with major public health implications, such as arthritis, diabetes, neurodegenerative diseases, atherosclerosis, and cancer (Bishop et al., 2007, Couchman, 2010, Mikami and Kitagawa, 2013).

**Figure 1:**
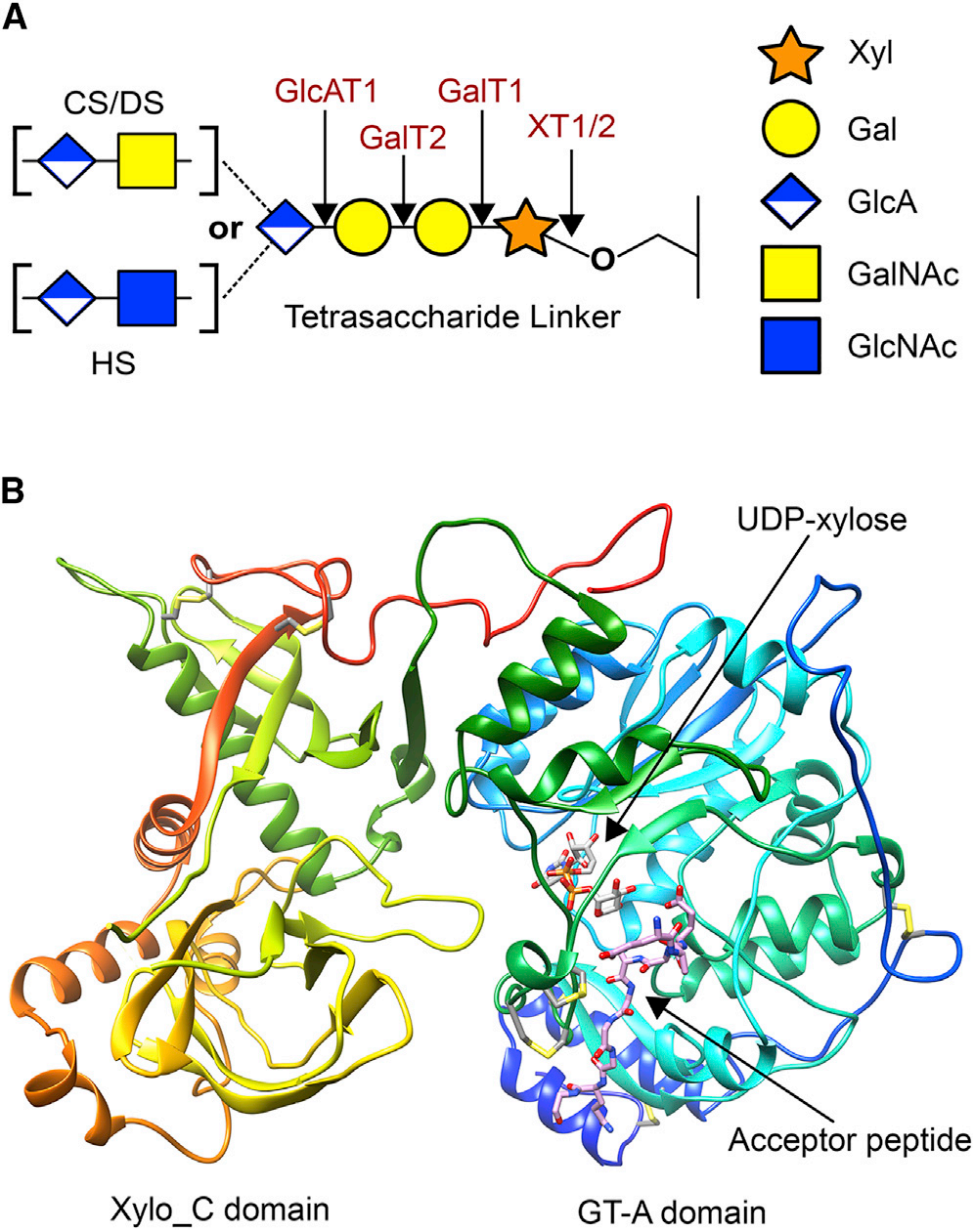
Crystal Structure of XT1 Complexed with UDP-Xylose and a Bikunin-Derived Acceptor Peptide (A) Schematic structure of the GAG tetrasaccharide linker. GTs involved in linker biosynthesis are indicated in red. The corresponding gene names are *XYLT1* (XT1), *XYLT2* (XT2), *B4GALT7* (GalT1), *B3GALT6* (GalT2), and *B3GAT3* (GlcAT1). (B) Crystal structure of human XT1, colored from N terminus (blue) to C terminus (red). UDP-xylose (silver) and peptide **2** (pink) are shown in stick representation, as are the disulfide bonds. See also Figure S1.

The GAG component of PGs is synthesized in the Golgi compartment during passage of the core protein through the secretory pathway. HS, CS, and DS are attached to serine residues on the core protein through a common GlcA-β1,3-Gal-β1,3-Gal-β1,4-Xyl-β1-*O*-Ser tetrasaccharide linker (Kreuger and Kjellen, 2012, Mikami and Kitagawa, 2013). The linker is synthesized by four glycosyltransferases (GTs) acting sequentially (Figure 1A); phosphorylation of the xylose by Fam20B is required for addition of the second galactose (Koike et al., 2009, Koike et al., 2014, Wen et al., 2014). The first enzyme in the pathway, peptide *O*-xylosyltransferase (XT, EC 2.4.2.26), catalyzes the transfer of xylose from uridine diphosphate (UDP)-α-D-xylose onto serine and thus determines the site(s) of GAG attachment on the core protein. The serine-linked xylose is in β-anomeric configuration and XT is therefore classified as an “inverting” GT (Lairson et al., 2008). *Caenorhabditis elegans* and *Drosophila melanogaster* have one XT (known as sqv-6 and OXT, respectively), whereas vertebrates have two isozymes, XT1 and XT2 (60% amino acid identity in humans) (Götting et al., 2000, Hwang et al., 2003, Wilson, 2002). Disruption of tetrasaccharide linker biosynthesis in mice by genetic ablation of the glucuronyl transferase results in embryonic lethality before the 8-cell stage (Izumikawa et al., 2010). The combined function of XT1 and XT2 is expected to be similarly essential, but double-knockout mice have not been described. Genetic screens in zebrafish and mouse have revealed a function of XT1 in chondrocyte maturation during bone development (Eames et al., 2011, Mis et al., 2014). XT2-deficient mice are viable, but develop polycystic liver and kidney disease (Condac et al., 2007). In humans, *XYLT1* and *XYLT2* mutations cause two rare diseases with skeletal abnormalities, Desbuquois dysplasia type 2 and spondylo-ocular syndrome, respectively (Bui et al., 2014, Munns et al., 2015). The phenotypes suggest that XT1 and XT2 are not fully redundant, consistent with their somewhat different expression patterns (Eames et al., 2011, Roch et al., 2010).

XT1 and XT2 are type II transmembrane proteins consisting of a short amino-terminal region facing the cytosol, a single transmembrane helix, a stem region required for Golgi localization (Schön et al., 2006), a catalytic GT-A domain (Lairson et al., 2008, Müller et al., 2006), and a unique C-terminal domain of unknown function, termed “Xylo_C” in the Pfam database (Finn et al., 2008). This topology places the catalytic GT domain inside the Golgi lumen.

The acceptor peptide specificities of XT1 and XT2 have been inferred from the sequences of known GAG attachment sites: the acceptor serine generally is flanked by glycines, and there frequently is a patch of acidic residues between positions −4 and −2 (position 0 being the acceptor serine) (Esko and Zhang, 1996). These preferences were largely confirmed by a study using recombinant enzymes and peptide substrates (Roch et al., 2010). How the XT active site specifically selects certain serine residues for covalent modification has remained unknown, however.

To better understand the initial step of GAG biosynthesis, we have determined crystal structures of human XT1 complexed with UDP-xylose and various PG-derived acceptor peptides. Combined with biochemical results, the structures define the catalytic mechanism of XT1 and the molecular basis for selection of GAG attachment sites.

## Results

### XT1 Crystal Structure

We obtained crystals of a human XT1 construct spanning residues 232–959 and determined its structure at 1.9-Å resolution. Inspection of the GT active site revealed patchy electron density for a co-purified, unidentified ligand. To obtain defined complexes, we used crystal soaking to replace the co-purified ligand with dodecapeptides derived from the PGs bikunin and syndecan 1. We also prepared a ternary complex of XT1 with the sugar donor, UDP-xylose, and an inactive bikunin-derived peptide in which the acceptor serine was replaced by alanine (Table 1). In total, we determined nine crystal structures at resolution limits of 1.9–2.7 Å (Table 2). Except for the ligands and their immediate surroundings the structures are very similar, and the following description of the structure is based on the ternary complex with UDP-xylose and peptide **2** (2.0 Å resolution).

**Table 1:** Peptides Used in this Study

Peptide	Sequence
1	QEEEG**S**GGGQGG
2	QEEEGAGGGQGG
3	QEPEG**S**GGGQGG
4	QEEEY**S**GGGQGG
5	QEEEG**S**AGGQGG
6	QEEEG**S**GVGQGG
7	QEEEG**S**GGPQGG
8	PAAEG**S**GEQDFT

Peptides **1** and its variants (**2** to **7**) are derived from bikunin. Peptide **8** is derived from syndecan 1. Acceptor serines (S215 in bikunin, S206 in syndecan 1) are in bold.

**Table 2:** Crystallographic Statistics

	Apo XT1	XT1 + Peptide 1	XT1 + Peptide 2 + UDP-Xylose	XT1 + Peptide 3	XT1 + Peptide 4	XT1 + Peptide 5	XT1 + Peptide 6	XT1 + Peptide 7	XT1 + Peptide 8
Data Collection									

Space group	*P*2_1_2_1_2_1_	*P*2_1_2_1_2_1_	*P*2_1_2_1_2_1_	*P*2_1_2_1_2_1_	*P*2_1_2_1_2_1_	*P*2_1_2_1_2_1_	*P*2_1_2_1_2_1_	*P*2_1_2_1_2_1_	*P*2_1_2_1_2_1_
Cell dimensions									
*a* (Å)	67.28	67.31	67.46	67.38	66.56	67.47	67.63	67.54	67.28
*b* (Å)	86.78	86.68	86.89	86.82	85.96	86.69	86.90	86.35	86.72
*c* (Å)	153.25	152.85	152.91	153.20	151.22	152.64	152.39	153.63	153.47
Resolution (Å)	76.63–1.87 (1.94–1.87)	53.16–2.09 (2.17–2.09)	61.72–2.00 (2.07–2.00)	75.53–2.02 (2.09–2.02)	40.18–1.94 (2.01–1.94)	76.32–2.56 (2.65–2.56)	61.81–2.06 (2.13–2.06)	57.68–2.69 (2.78–2.69)	61.62–2.43 (2.52–2.43)
CC_1/2_	0.994 (0.139)	0.996 (0.627)	0.996 (0.640)	0.997 (0.742)	0.998 (0.602)	0.945 (0.331)	0.980 (0.256)	0.949 (0.323)	0.996 (0.572)
*R*_merge_	0.242 (3.012)	0.134 (1.235)	0.183 (1.824)	0.107 (1.130)	0.076 (1.164)	0.297 (1.868)	0.256 (1.694)	0.288 (1.383)	0.120 (1.048)
*I*/σ(*I*)	8.6 (1.0)	8.9 (1.3)	9.1 (1.3)	10.9 (1.5)	8.7 (1.2)	4.5 (1.1)	5.3 (1.2)	4.1 (1.4)	9.4 (1.5)
Completeness (%)	99.2 (95.8)	99.6 (99.9)	99.9 (99.9)	99.0 (99.1)	98.7 (99.4)	99.1 (94.6)	97.4 (93.9)	99.4 (94.2)	98.4 (99.2)
Multiplicity	7.0 (6.0)	5.5 (5.8)	6.5 (6.7)	4.3 (4.4)	4.2 (4.2)	4.2 (4.0)	4.3 (4.4)	4.3 (4.1)	4.3 (4.4)

Refinement									

No. of reflections	74,376	53,547	61,400	59,105	64,174	29,426	55,102	25,946	34,054
*R*_work_/*R*_free_	0.195/0.222	0.206/0.227	0.199/0.229	0.204/0.225	0.185/0.212	0.238/0.279	0.242/0.272	0.240/0.279	0.197/0.235
No. of atoms									
Proteins	5,513	5,597	5,688	5,537	5,502	5,553	5,552	5,473	5,508
Ligands + solvent	485	293	533	263	274	70	117	60	172
RMSDs									
Bond lengths (Å)	0.005	0.004	0.003	0.003	0.004	0.002	0.002	0.006	0.003
Bond angles (°)	1.04	0.60	0.53	0.57	0.64	0.48	0.49	0.87	0.51
Ramachandran plot									
Favored (%)	98.4	97.4	98.3	97.7	97.7	96.3	97.3	97.8	97.8
Outliers (%)	0	0	0	0	0	0	0	0.3	0

XT1 has a two-lobed structure, with one lobe comprising the catalytic GT-A domain and the other comprising the Xylo_C domain (Figure 1B). The GT-A domain of XT1 (residues 325–620) is most similar to that of leukocyte-type core 2 β1,6-*N*-acetylglucosaminyltransferase (C2GnT-L) (Pak et al., 2006) (root-mean-square deviation [RMSD] of 1.7 Å over 252 Cα atoms, sequence identity 27%). The most striking differences between XT1 and C2GnT-L are the path of the N-terminal extension (Figure S1A) and the active-site entrance, which is open in C2GnT-L but covered by a flap in XT1 (residues 555–575) (Figure S1B). In XT1, the ∼90 residues preceding the GT-A domain loop around the back of the domain, placing the two N-terminal α helices on the opposite side of the active-site cleft compared with C2GnT-L. The two helices are anchored to the GT-A domain by a disulfide bond (C276-C461); a second disulfide bond (C257-C261) staples the N terminus to the first helix. An arginine in the first helix, R270, forms a conspicuous salt bridge with D570 at the tip of the active-site flap. The flap itself is an irregular hairpin containing two disulfide bonds (C561-C574 and C563-C572) and two aromatic residues involved in interactions with the GT-A domain (Y565 and W571). The returning strand in the hairpin forms main-chain interactions with the acceptor peptide (see below) and also contacts the sugar donor via S575. The equivalent loop in C2GnT-L adopts a very different conformation, but also participates in donor and acceptor recognition (Pak et al., 2006, Pak et al., 2011).

The Xylo_C domain consists of a cystatin-like fold (residues 644–722 and 915–929), into which is inserted an immunoglobulin (Ig)-like fold (residues 723–840) and an α-helical region (residues 841–914) (Figure S1C). Because these elements share a continuous hydrophobic core, the Xylo_C domain is best described as a single domain. The closest approach between the Xylo_C domain and the GT-A active site is made by the extended loop between the second and third strands of the Ig fold (residues 744–766) (Figure 1B). The tip of this loop makes several interactions with the active-site flap, including a prominent salt bridge between R754 and E601. There are two disulfide bonds in the Xylo_C domain (C675-C927 and C920-C933), which appear to stabilize the conformation of the C-terminal segment of XT1. The last 15 residues make a return to the GT-A domain and interact extensively with it via two conserved leucines (L949 and L958) and two conserved arginines (R957 and R959). The Xylo_C domain contains the only N-linked glycosylation site in our structure at N777; the other potential site at N421 does not appear to be glycosylated.

### UDP-Xylose Binding Site

**Figure 2:**
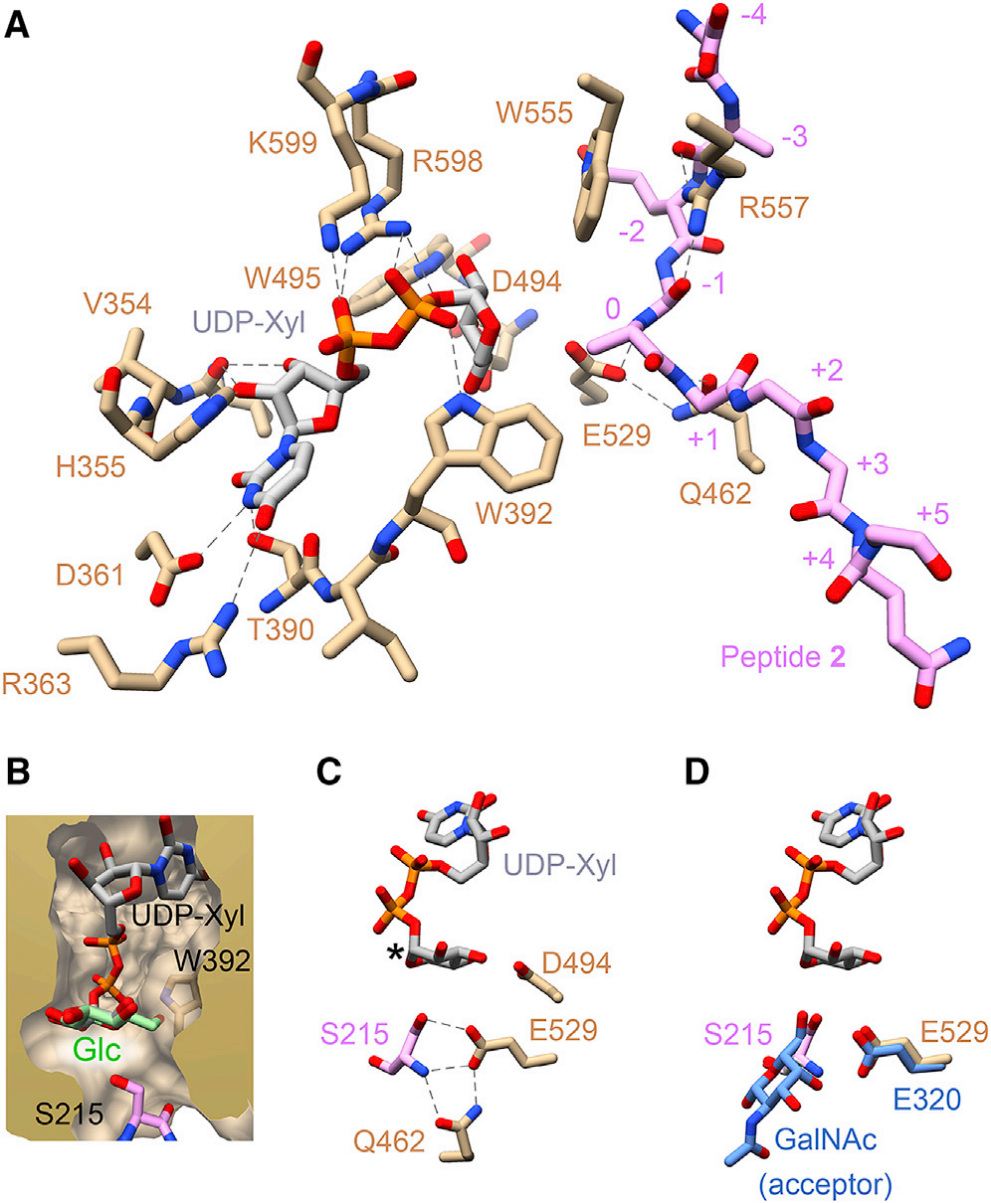
Active Site of XT1 (A) Close-up view of the active site in the ternary Michaelis-like complex with UDP-xylose (silver) and peptide **2** (pink). Selected XT1 residues are shown in gold. Hydrogen bonds are shown as dashed lines. (B) Surface representation of the UDP-xylose binding pocket. A glucose monosaccharide (pale green) is superimposed with the xylose group to highlight the clash of the C6-OH group with W392. (C) Key catalytic residues in the XT1 active site. The active peptide **1** was superimposed with the inactive peptide **2** to obtain a model of the Michaelis complex. The xylose C1 atom is indicated by an asterisk. (D) Superposition of the active site of XT1 with the C2GnT-L complex with the acceptor Gal-β1,3-GalNAc (PDB: 2GAK, blue). See also Figure S2.

Metal-ion-dependent GTs typically have a DXD sequence motif; the two aspartic acids coordinate a manganese ion, which in turn binds to the diphosphate moiety of the UDP-sugar donor (Lairson et al., 2008). XT1 contains two conserved DXD motifs (residues 314–316 and 745–747, respectively), but neither of them is located close to the UDP-xylose binding site. Instead of interacting with a manganese ion, the UDP diphosphate moiety interacts with two positively charged XT1 residues, R598 and K599, which are joined by a rare *cis*-peptide bond (Figure 2A). In C2GnT-L, which like XT1 is a metal-ion-independent GT, the UDP diphosphate moiety is also bound by two positively charged residues, but they are contributed by different GT-A regions (R378 and K401) (Pak et al., 2011).

The complex of XT1 with UDP-xylose was obtained with the inactive peptide **2**, in which the acceptor serine is mutated to alanine. Electron density for the xylose group was visible (Figure S2A) and refinement yielded a ^4^C_1_ chair conformation for the xylose group. However, residual difference density around the xylose and β-phosphate group, and the high *B* factors of the xylose atoms, suggest a mixed population of conformations or partial hydrolysis of UDP-xylose. Similar disorder of the sugar donor has been observed in other GTs (Lazarus et al., 2011, Vrielink et al., 1994). The xylose group forms polar contacts with D494 and E529, and apolar contacts with W392 and W495 (Figure 2A). Of particular note is the close contact between the xylose C5 atom and the side chain of W392, which likely prevents binding of larger hexose and hexosamine sugars (Figure 2B).

Superposition of the binary complex with active peptide **1** and the ternary complex with inactive peptide **2** allowed us to construct a model of the Michaelis complex. In this model, the serine OH group is perfectly positioned for nucleophilic attack on the UDP-xylose C1 carbon atom (Figure 2C). When the XT1 complex with peptide **1** is compared with the C2GnT-L complex with the acceptor Gal-β1,3-GalNAc (Pak et al., 2006), the attacking oxygen atoms occupy the same position (Figure 2D). Thus, we believe that we have crystallized a catalytically competent conformation of XT1. Indeed, when we soaked XT1 crystals with UDP-xylose and catalytically active peptide **1**, the electron density for the xylose group disappeared, presumably due to GT activity within the crystal (not shown).

### Peptide Binding Site

**Figure 3:**
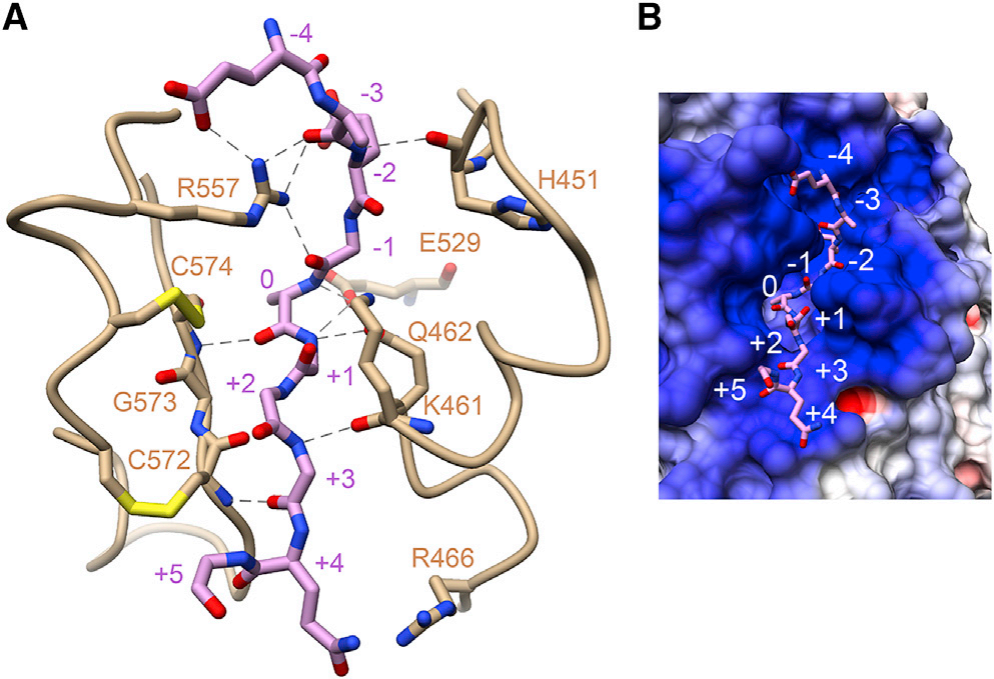
Peptide Binding Site of XT1 (A) Peptide **2** (pink) binds in a cleft between two surface loops of XT1. Selected XT1 residues are shown in gold, including the presumed catalytic base, E529. Hydrogen bonds are shown as dashed lines. (B) Electrostatic surface representation of the peptide binding pocket of XT1 (blue, positive potential; red, negative potential). See also Figure S2.

In the ternary complex, continuous electron density was observed for 9 of the 12 amino acid residues of peptide **2** (Figure S2B). The peptide is bound within a cleft delimited on one side by the disulfide-bonded hairpin formed by residues 555–575, and on the other side by a loop containing a short α helix, the last turn of which constricts the width of the cleft (XT1 residues K461 and Q462) (Figure 3A). It is noteworthy that all but one of the 11 hydrogen bonds between peptide **2** and the GT-A domain involve the peptide's main-chain carbonyl and amide groups, i.e., they are not sequence specific. However, the acidic residues at positions −2 to −4 are located favorably in a positively charged environment (Figure 3B). The active peptide **1** is bound in an identical manner. The peptide conformation is relatively extended but bulges at the position of the acceptor serine, as if the side chains of Q462 and E529 were pushing the acceptor serine toward the sugar donor (Figure 2A).

### Validation by Site-Directed Mutagenesis

**Figure 4:**
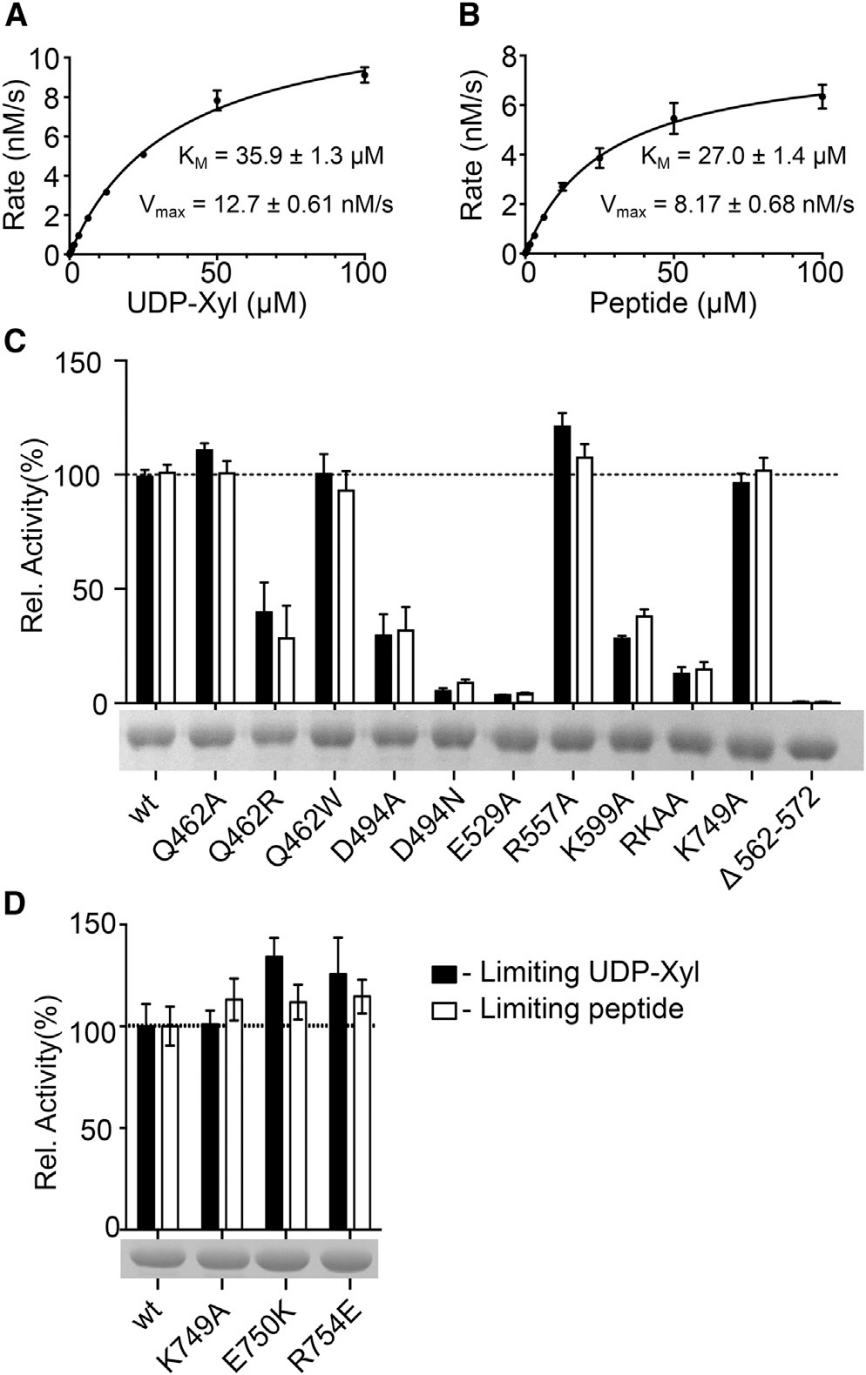
Enzymatic Analysis of XT1 and Its Mutants (A) Michaelis-Menten plot for UDP-xylose at 100 μM peptide **1**. (B) Michaelis-Menten plot for peptide **1** at 100 μM UDP-xylose. (C and D) Enzymatic activity of XT1 mutants (filled bars: 10 μM UDP-xylose, 100 μM peptide **1**; open bars: 100 μM UDP-xylose, 10 μM peptide **1**). Slices of Coomassie blue-stained reducing SDS-PAGE gels of the mutants are shown below the graphs. RKAA denotes the R598A/K599A double mutant. Error bars represent the SD of triplicate data points in a representative experiment. All experiments were carried out three times.

We tested our XT1 construct for enzymatic activity by measuring UDP release at a range of donor and acceptor concentrations (Figures 4A and 4B); the K_M_ values derived from this analysis are in good agreement with those previously reported (Roch et al., 2010). We then used the activity assay to probe the role of selected conserved active-site residues. Superposition of acceptor complexes of XT1 and C2GnT-L (Figure 2D) highlights a conserved glutamic acid (E529 and E320, respectively). Given that a similarly positioned acidic residue acts as the catalytic base in many inverting GTs with a GT-A fold (Lairson et al., 2008), we hypothesized that E529 in XT1 may deprotonate the serine for nucleophilic attack on the UDP-xylose C1 atom. Mutation of E529 to alanine (the E529Q mutant was not secreted by the HEK293 cells) indeed abolished enzymatic activity (Figure 4C). Mutation of residues involved in UDP-xylose binding (D494, R598, and K599) also dramatically reduced enzyme activity. Individual mutations of residues involved in acceptor peptide binding (Q462 and R557) had little effect, but shortening of the hairpin that sits over the active site (Δ563-572 mutant) abolished activity, validating its essential role in acceptor peptide binding. Mutations of the few Xylo_C residues interacting with the GT-A domain (K749A, E750K, and R754E) had no effect (Figure 4D), suggesting that the Xylo_C domain does not contribute to catalysis. All attempts to produce soluble XT1 variants with a shortened 744–766 loop or lacking the entire Xylo_C domain were unsuccessful, suggesting that the Xylo_C domain is required for XT1 folding or secretion.

### Specificity of Acceptor Recognition

**Figure 5:**
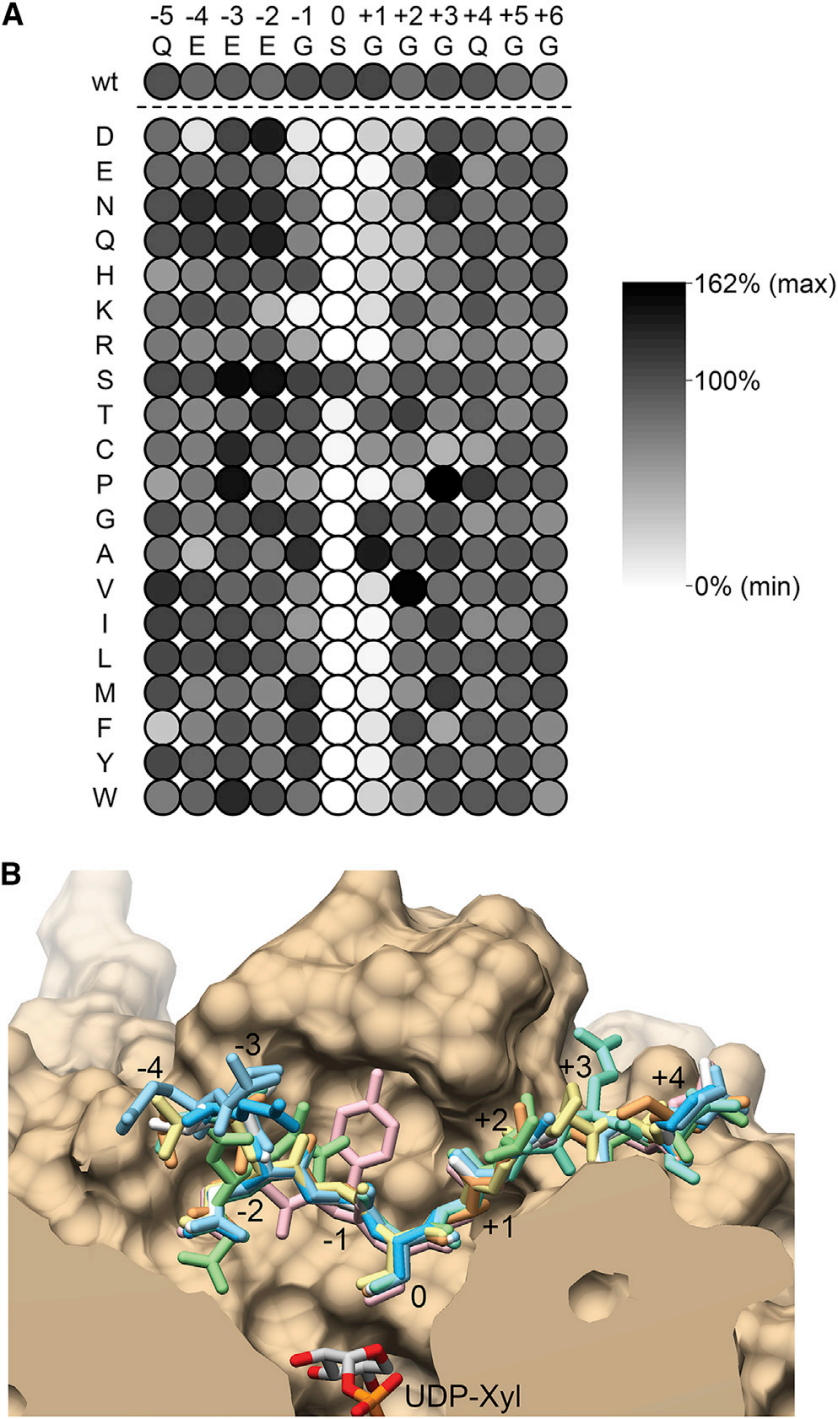
Acceptor Specificity of XT1 (A) XT1 activity toward a bikunin-derived peptide library. Single amino acids in the wild-type sequence were substituted with every one of the 20 amino acids, grouped by amino acid type, as indicated. The wild-type sequence occurs 12 times in this array and the activity toward these peptides is shown in the top row. Luminescence was normalized against UDP standards and converted into gray-scale values. The experiment was carried out three times, and mean values are shown. (B) Superposition of the eight peptide complexes of XT1. Shown is a cut-through of the XT1 peptide binding pocket, with the peptides in stick representation (**1**, white; **2**, dark blue; **3**, light blue; **4**, pink; **5**, orange; **6**, light green; **7**, yellow; **8**, cyan). The N terminus of the peptides is on the left. The modified serine (position 0) is situated above the UDP-xylose donor (silver). See also Figure S3.

To explore the acceptor specificity of XT1, we interrogated a peptide library based on the bikunin acceptor sequence. Each position in peptide **1** was substituted with each of the 20 amino acids, yielding a 12 × 20 array. Activity assays with these 240 peptides revealed that XT1 is surprisingly unspecific (Figure 5A), consistent with our observation that most contacts between XT1 and the acceptor peptide are mediated by the acceptor backbone (Figure 3A). As expected, a serine is required at position 0 (the xylosylation site), although the activity with an acceptor threonine was noted to be above background (7% of wild-type). Position +1 has a strong preference for small amino acids (G, A, S, T, C; note that serine may act as an alternative acceptor). In contrast, position −1 has a wide tolerance for uncharged amino acids, accommodating even large aromatic residues such as tryptophan. The +2 position exhibited an unexpected preference for the β-branched amino acid valine (and, to a lesser extent, threonine). Proline can be accommodated at positions −3 and +3, where the wild-type peptide naturally adopts a backbone conformation favorable for proline. Individual substitution of the glutamic acids at positions −2, −3, and −4 had little effect, suggesting that the frequent concentration of acidic residues upstream of GAG attachment sites is due to non-specific electrostatic interactions with the positively charged peptide binding cleft (Figure 3B).

To visualize how the different amino acid substitutions are accommodated in the peptide binding cleft, we selected five variants of the bikunin-derived peptide for structure analysis (Table 1, peptides **3–7**). Three bikunin variants bound identically to the wild-type peptide: peptide **3** (proline at position −3), peptide **5** (alanine at position +1), and peptide **7** (proline at position +3). The substitution of glycine by valine at position +2 (peptide **6**) results in a 72° rotation of the 217-218 peptide bond, which allows K461 to form a new hydrogen bond with the backbone carbonyl of the peptide at the −2 position. This induces a kink in the peptide, pulling the glutamic acid side chain at position −2 out of the binding site and replacing it with that of the glutamic acid at position −3 (Figure 5B). To accommodate this rearrangement the 449–466 loop moves slightly, widening the peptide binding cleft by ∼3 Å. The substitution of glycine by tyrosine at position −1 (peptide **4**) causes a similar widening of the peptide binding cleft (Figure 5B). The tyrosine side chain packs against the side chain of F498, displacing it by ∼1 Å. This is accompanied by a flip of the peptide bond between positions −1 and −2 of the acceptor peptide, which, however, does not affect the conformation of the glutamic acid side chain at position −2. Finally, we also determined the structure of XT1 with a peptide derived from syndecan 1 (Table 1, peptide **8**). Peptide **8** also bound identically to the bikunin-derived peptide **1**. Both peptides have glutamic acids at position −2, and their side chains assume identical conformations. The side chain of the glutamic acid at position +2 of peptide **8** is not visible in the electron density, whereas the side chain of the glutamine at position +3 is well ordered and packs against W571. From these experiments, we conclude that the peptide binding site of XT1 is promiscuous, except for the requirement of a small amino acid side chain at the position following the acceptor serine. These findings are in good agreement with the sequences of known GAG attachment sites (Figure S3).

### Effects of Disease-Causing Mutations

The *pug* dwarf mutant mouse has a missense mutation in *Xylt1* (W932R) that leads to a ∼80% reduction in XT1 activity in cell culture and a corresponding reduction of cartilage PGs (Mis et al., 2014). W932 is conserved in all vertebrate and invertebrate Xylo_C domains and mediates a prominent apolar contact of the C-terminal segment to the cystatin-like domain. Chondrocytes from *pug* mice show reduced Xylt1 staining in the *cis-*Golgi (Mis et al., 2014), suggesting that the phenotype is due to protein misfolding rather than impaired catalytic activity. The few *XYLT1* missense mutations in human patients (R481W and R598C) have been shown to cause ER retention of the mutated proteins (Al-Jezawi et al., 2017); the more frequent frameshift or exon-skipping mutations (Bui et al., 2014, Schreml et al., 2014) are also predicted to result in misfolded protein.

## Discussion

We have determined the first structure of a peptide *O*-xylosyltransferase and have revealed how this enzyme selects serine residues for GAG attachment. Besides the expected GT-A domain, our XT1 structure displays several features not seen in other GTs: (1) a *cis*-bonded arginine-lysine pair that interacts with the diphosphate moiety of the UDP-sugar donor; (2) an active-site flap that plays a prominent role in acceptor peptide binding; and (3) a non-catalytic Xylo_C domain. The structure of the Michaelis-like ternary complex with UDP-xylose and peptide **2** suggests that XT1 follows a direct-displacement S_N_2-like mechanism, similarly to other inverting GTs with a GT-A fold (Lairson et al., 2008). The specificity for UDP-xylose as a donor is likely to be stringent, given the restrictive shape of the xylose binding pocket (Figure 2B). In contrast, we show that the specificity for the peptide acceptor is lax, except for the identity of the modified residue and its immediate neighbor in the +1 position (Figure 5A). Transfer onto serine is overwhelmingly preferred, but there is measurable activity when a threonine residue is present instead. The greatly reduced activity toward threonine acceptors likely results from steric clashes of the threonine γ-methyl group with the side chain of W392 and the XT1 main chain at position 574. No threonine-linked PGs have ever been found, but our activity data suggest that their existence cannot be ruled out. The limited range of amino acids permitted at the position +1 is readily explained by the shape of the peptide binding cavity, which features a narrow constriction on the C-terminal side of the acceptor serine. The structure with peptide **5** shows that the extra methyl group of alanine can be accommodated at this position, but larger side chains would prevent the acceptor serine OH group from reaching the xylose C1 atom. Curiously, all but one of the known GAG attachment sites have a glycine in position +1 (Figure S3), even though XT1 works equally well with glycine or alanine (Figure 5A). One explanation for this discrepancy could be that a subsequent step in GAG biosynthesis has a more stringent requirement for glycine in position +1. The most likely candidate is phosphorylation of the xylose O2 atom by Fam20B (Koike et al., 2009, Wen et al., 2014), which might be impeded by any side chain in position +1. If XT1 indeed xylosylates more sites than are eventually elongated, the incomplete capped linkers (Mikami and Kitagawa, 2013, Wen et al., 2014) may be degraded or they may have escaped detection because of their small size. It is also important to remember that the sequence of the acceptor site is not the only determinant of GAG attachment. Because XT1 acts on folded proteins in the *cis*-Golgi compartment, the accessibility of the acceptor serine is a major factor. From our structures, we estimate that at least four residues either side of the serine need to be in an extended conformation to fit into the active-site cleft of XT1. Indeed, most known GAG attachment sites occur either in predicted surface loops or in stretches of unstructured polypeptide. The role of the unique active-site flap in XT1 may be to provide a β-strand-like interface for the extended peptide substrate and to shape the constriction at position +1.

The presence of a Xylo_C domain is a hallmark of XTs involved in GAG biosynthesis; the domain is not found in other XTs (Li et al., 2017, Yu et al., 2015, Yu et al., 2016). The role of the Xylo_C domain remains unclear. We think it unlikely that the Xylo_C domain is required for catalysis, given that mutations in the 744–766 loop, which mediates the only direct contact between the Xylo_C and GT-A domains, did not impair xylose transfer (Figure 4D). It is possible that the Xylo_C domain helps to recruit one or several of the enzymes carrying out the subsequent steps in GAG biosynthesis, analogous to the formation of GT complexes in the biosynthesis of other glycans (Oikawa et al., 2013). As previously discussed, we believe that our XT1 structure represents a catalytically competent conformation. However, the relative orientation of the GT-A and Xylo_C domains may well change to facilitate substrate binding or product release.

Reversible modification of serines and threonines by GlcNAc is a widespread and important post-translational modification of intracellular proteins (Bond and Hanover, 2015). The reaction is carried out by a single *O*-GlcNAc transferase (OGT), which modifies hundreds of different targets. The structural basis for OGT promiscuity has been revealed by crystallographic studies (Lazarus et al., 2011, Lazarus et al., 2012, Pathak et al., 2015, Schimpl et al., 2012). OGT belongs to the GT-B superfamily and has an N-terminal non-catalytic domain consisting of 13.5 tetratricopeptide repeats. Analysis of ternary complexes showed that the acceptor peptide binds over the UDP-GlcNAc pocket, thereby blocking access to it. OGT thus follows an ordered “bi-bi” mechanism, in which UDP-GlcNAc binds before the peptide and UDP leaves after the modified peptide. In XT1, by contrast, the peptide and UDP-Xyl have independent routes of access in the ternary complex, suggesting that they can exchange independently. Like in XT1, the acceptor peptides of OGT are bound in an extended conformation. The OGT recognition sequence is degenerate, but there is a general preference for β-branched amino acids and prolines flanking the modified serine/threonine (Pathak et al., 2015). This contrasts with XT1's strict requirement for a glycine or alanine in the +1 position. Indeed, the peptide binding cleft in OGT is less severely constricted at the +1 position than in XT1.

In conclusion, our study has provided detailed mechanistic insight into the initiation of GAG biosynthesis by showing how the unique active-site architecture of XT1 selects particular serine residues for xylosylation. A major aim for future studies will be to understand whether the sequence context of the modified serine plays a role in determining the nature of the elongated GAG chain.

## STAR★Methods

### Key Resources Table

**Table:** 

REAGENT or RESOURCE	SOURCE	IDENTIFIER
**Bacterial and Virus Strains**		

*E*. *coli* DH5α	Invitrogen	Cat# 18265017

**Chemicals**, **Peptides**, **and Recombinant Proteins**		

UDP-xylose	Carbosource	https:www.ccrc.uga.edu/∼carbosource/css/xglose.htm
Various synthetic peptides (Table 1, this paper)	Genscript	N/A
Morpheus screen condition C10	Molecular Dimensions	MDSR-47-C10

**Critical Commercial Assays**		

UDP-Glo glycosyltransferase kit	Promega	Cat# V6961

**Deposited Data**		

Crystal structure, Apo XT1	This paper	PDB: 6FOA
Crystal structure, XT1 + peptide 1	This paper	PDB: 6EJ8
Crystal structure, XT1 + peptide 2 + UDP-xylose	This paper	PDB: 6EJ7
Crystal structure, XT1 + peptide 3	This paper	PDB: 6EJ9
Crystal structure, XT1 + peptide 4	This paper	PDB: 6EJA
Crystal structure, XT1 +peptide 5	This paper	PDB: 6EJB
Crystal structure, XT1 +peptide 6	This paper	PDB: 6EJC
Crystal structure, XT1 +peptide 7	This paper	PDB: 6EJD
Crystal structure, XT1 +peptide 8	This paper	PDB: 6EJE

**Experimental Models**: **Cell Lines**		

HEK293-F	Thermo Fisher	R79007

**Oligonucleotides**		

PCR amplification primers for XT1 construct: 5’-CGGAATTCGCTAGCCGATGTGTCCAGACCGCCT 5’-AGGATCCGCGGCCGCCCTGAGCCGGCCATCAGG	Life Technologies	N/A

**Recombinant DNA**		

Human XT1 cDNA	Dharmacon	BC045778 Clone ID: 4791553
Plasmid pCEP-Pu-N-His-TEV	Pulido et al., 2017	N/A

**Software and Algorithms**		

XIA2	Winter et al., 2013	https://xia2.github.io/index.html
XDS	Kabsch, 2010	http://xds.mpimf-heidelberg.mpg.de/
DIALS	Waterman et al., 2016	https://dials.github.io/index.html
AIMLESS	Evans and Murshudov, 2013	http://www.ccp4.ac.uk/html/aimless.html
CTRUNCATE	Winn et al., 2011	http://www.ccp4.ac.uk/html/ctruncate.html
POINTLESS	Evans, 2011	http://www.ccp4.ac.uk/html/pointless.html
PHASER	McCoy et al., 2007	http://www.phaser.cimr.cam.ac.uk/index.php/Phaser_Crystallographic_Software
PHENIX	Adams et al., 2010	https://www.phenix-online.org/
COOT	Emsley and Cowtan, 2004	https://www2.mrc-lmb.cam.ac.uk/personal/pemsley/coot/
UCSF Chimera	Pettersen et al., 2004	https://www.cgl.ucsf.edu/chimera/

### Contact for Reagents and Resource Sharing

Further information and requests for resources and reagents should be directed to and will be fulfilled by the Lead Contact, Erhard Hohenester (e.hohenester@imperial.ac.uk).

### Experimental Model and Subject Details

#### HEK293 Cell Culture

HEK293-F cells were grown in FreeStyle medium at 37°C, 8% CO_2_.

### Method Details

#### Protein Production

DNA coding for residues 232-959 of human XT1 was amplified from a cDNA clone (Dharmacon) using Q5 polymerase (New England Biolabs) and ligated into a modified pCEP-Pu vector that adds a TEV protease-cleavable His-tag at the N-terminus of the secreted protein (Pulido et al., 2017). The vector was transfected into FreeStyle 293-F cells (Thermo Fisher Scientific) using linear polyethylimine (MW 25,000; Polysciences). The cell culture supernatant was harvested after 5 days of shaking the cells at 120 rpm, 37°C, 8% CO_2_. The filtered conditioned medium was adjusted to pH 7.5 by the addition of 1 M HEPES pH 7.5 to a final concentration of 20 mM, and loaded onto a 1 ml HisTrap Excel column (GE Healthcare) at 4°C. The bound protein was eluted with a 0-500 mM imidazole gradient over 30 column volumes. Fractions containing XT1 were pooled and dialysed overnight against 50 mM Tris, 2 mM EDTA, pH 8.5. This material was then loaded onto a 5 ml HiTrap Heparin HP affinity column (GE Healthcare) and eluted using a 0-1 M NaCl gradient over 30 column volumes. XT1 eluted at a NaCl concentration of 480 mM. Fractions containing XT1 were pooled, concentrated to 5 mg/ml, aliquoted, snap-frozen in liquid nitrogen, and stored at -80°C. Prior to use in assays and crystallisation screens, this material was further purified by size exclusion chromatography using a Superdex 200 Increase column (GE Healthcare) equilibrated in 20 mM Tris, 130 mM NaCl, pH 7.5. The final yield was approximately 5 mg of XT1 protein per litre of cell culture supernatant. Mutations were introduced either using the QuikChange XL-II site-directed mutagenesis kit (Agilent) or overlap extension PCR.

#### Crystallisation

Crystals were obtained by hanging-drop vapour diffusion using a solution from the Morpheus screen (Molecular Dimensions). 1 μl of a 6 mg/ml XT1 protein solution in 20 mM Tris, 130 mM NaCl, pH 7.5 was mixed with 1 μl of 55-65% precipitant mix 2 (PEG8000 and ethylene glycol), Bicine/Tris buffer at pH 7.5, and 0.1 M of a sodium nitrate/sodium phosphate/ammonium sulphate mix. Peptides (Genscript) and UDP-xylose (Carbosource) were soaked into the crystals either by sequential addition of ligand to the crystallisation drop (up to final concentrations of peptide and UDP-xylose of 0.5 and 3.5 mM, respectively), or by transfer of crystals to crystallisation buffer containing ligand (0.7 mM peptide, 3.5 mM UDP-xylose). The crystals were flash-frozen in liquid nitrogen for data collection.

#### Data Collection and Structure Determination

X-ray diffraction data were collected at the Diamond Light Source on beamlines I03 (λ = 0.9763 Å) and I04-1 (λ = 0.9282 Å). Automatic data processing was performed by DIALS (Waterman et al., 2016) or XIA2 (Winter et al., 2013), which make use of XDS (Kabsch, 2010), AIMLESS (Evans and Murshudov, 2013), CTRUNCATE (Winn et al., 2011) and POINTLESS (Evans, 2011). The apo XT1 structure was solved by molecular replacement using PHASER (McCoy et al., 2007), as implemented in PHENIX (Adams et al., 2010). A trimmed search model of the GT-A domain of C2GnT-L (PDB 2GAK), corresponding to ∼40% of the scattering matter in the asymmetric unit, was placed with a translation function Z-score of 9.0, and extended using a thorough auto-building and density modification protocol in PHENIX. When the protocol reached its cycle limit, ∼200 residues had been added and R_free_ reduced to 0.31. The remaining residues were readily built into positive difference electron density using COOT (Emsley and Cowtan, 2004). Refinement was carried out using PHENIX. The final model is complete except for the disordered N-terminus (purification tag and XT1 residues 232-251) and two surface loops (XT1 residues 310-313 and 730-735). No existing structures were used to guide the building of the Xylo_C domain. All complex structures were determined using the apo structure as an isomorphous phasing model. Data collection and refinement statistics are summarised in Table 2. The structure figures were generated using UCSF Chimera (Pettersen et al., 2004).

#### XT1 Enzyme Activity Assay

XT1 activity was determined by monitoring the release of UDP from UDP-xylose using a UDP-Glo glycosyltransferase assay kit (Promega). Enzyme and peptides were diluted in 50 mM Tris pH 7.0, 50 mM NaCl, and the reaction was started by the addition of UDP-xylose. The assay was carried out in white 96-well plates (Corning) using a reaction volume of 25 μl/well. After incubation at room temperature for 1 hour, the UDP detection step was carried following the manufacturer's instructions. The luminescence was read using a FLUOstar OPTIMA luminometer (BMG LABTECH). The activity of XT1 mutants was assayed at either 100 μM UDP-xylose and 10 μM peptide **1** (“limiting acceptor”) or 10 μM UDP-xylose and 100 μM peptide **1** (“limiting donor”). The peptide library assays were carried out with 100 μM UDP-xylose and 25 μM peptide. All assays were carried out in triplicate. Assays conducted across several 96-well plates were normalised to the signal of wells containing 50 μM UDP to correct for differences in the efficiency of the UDP detection. Curve fitting of kinetic data was carried out using GraphPad Prism.

### Data and Software Availability

Atomic coordinates and structure factors have been deposited in the Protein Data Bank under accession codes 6FOA (apo XT1), 6EJ8 (complex with peptide **1**), 6EJ7 (complex with peptide **2** and UDP-Xyl), 6EJ9 (complex with peptide **3**), 6EJA (complex with peptide **4**), 6EJB (complex with peptide **5**), 6EJC (complex with peptide **6**), 6EJD (complex with peptide **7**), 6EJE (complex with peptide **8**).

## Supplemental Information

Document S1. Figures S1–S3

Document S2. Article plus Supplemental Information
