# Supplementary material for: Structural Basis for the Initiation of Glycosaminoglycan Biosynthesis by Human Xylosyltransferase 1
Source: Structure. 2018 Jun 5;26(6):801–809.e3. doi: 10.1016/j.str.2018.03.014 (PMC5992326; doi:10.1016/j.str.2018.03.014)
Supplement: Document S1. Figures S1–S3 [file mmc1.pdf]

Structure, Volume *26*

Supplemental Information

Structural Basis for the Initiation of Glycosaminoglycan Biosynthesis by Human Xylosyltransferase 1

David C. Briggs and Erhard Hohenester

SUPPLEMENTAL INFORMATION

Structural basis for the initiation of glycosaminoglycan biosynthesis by human xylosyltransferase 1

David C. Briggs & Erhard Hohenester

Department of Life Sciences, Imperial College London, London SW7 2AZ, UK.

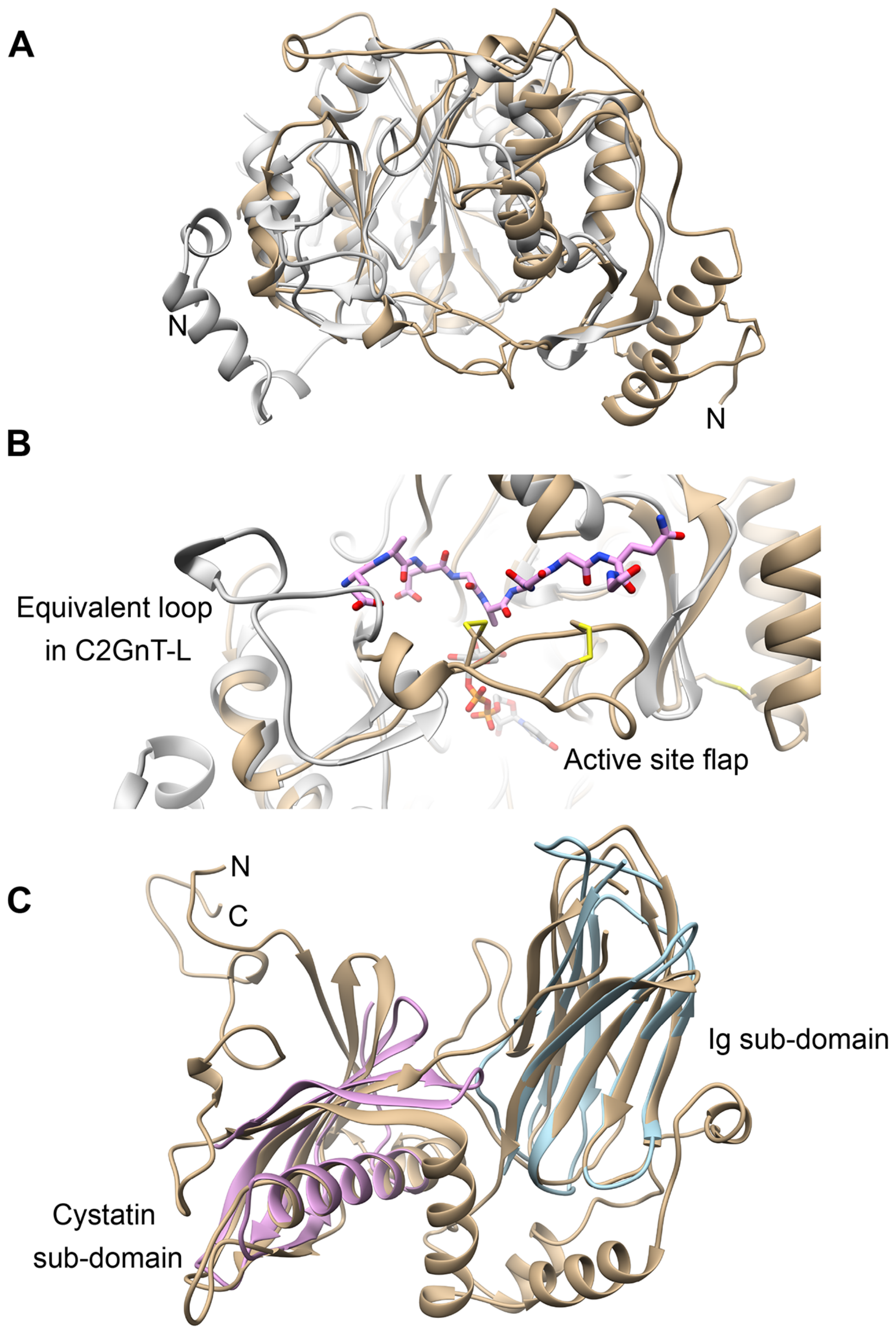

Figure S1–Related to Figure 1. Comparison of XT1 with other structures.
(A) Superposition of the GT-A domains of XT1 (gold) and C2GnT-L (PDB 2GAM, silver). The N-termini are labelled. (B) Close-up of the active sites of XT1 and C2GnT-L, showing the active site flap in XT1 and the radically different position of the equivalent loop in C2GnT-L. (C) Superposition of the XT1 Xylo_C domain (gold) with an Ig domain (PDB 2P9R, pale blue) and a cystatin domain (PDB 2W9P, pink).

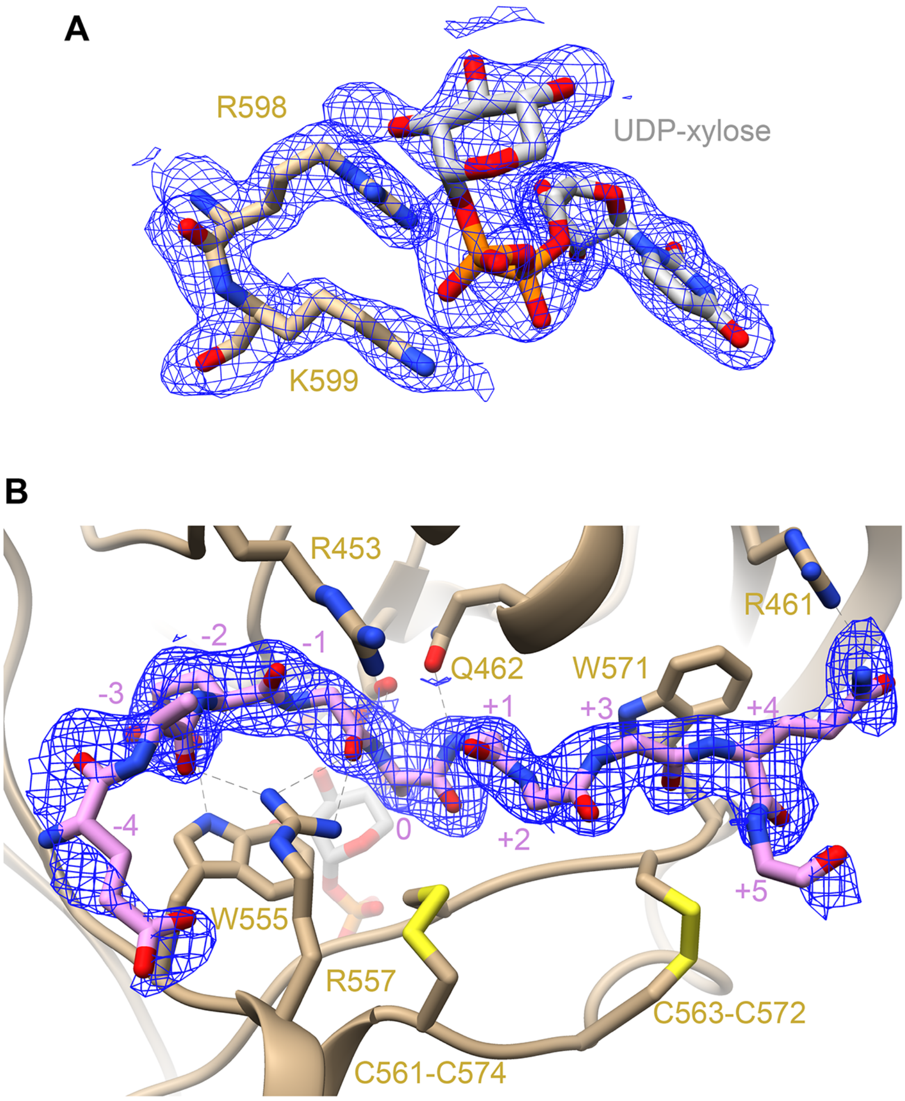

Figure S2–Related to Figure 2. Electron density of XT1 active site features. (A) UDP-xylose (silver) and the unusual *cis*-peptide bond between residues R598 and K599 (gold). (B) Peptide 2 (pink) and surrounding XT1 residues (gold). Shown are simulated annealing composite omit maps contoured at 1.5 σ.

Protein	Site	GAG	Sequence
AMBP		CS	QEEEGSGGGQLV
APP-L		CS	TTRPGSGLTNIK
BGN	1	MX	NDEEASGADTSG
BGN	2	MX	SGADTSGVLDPD
BGN	3	MX	PKGVFSGLRNMN
BGN	4	MX	NPLENSGFEPGA
COL12	1	CS	IPEYFSGPGTPL
COL12	2	CS	TALYASGAGDAL
COL12	3	CS	FATYSSGEGEPL
COL9		MX	QGLEGSADFLCP
CSPG4		CS	RQGESSGDMAWE
CSPG5		CS	TAEAGSGDAQAL
CXCR4		CS	TEEMGSGDYDSM
DCN		MX	LEDEASGIGPEV
EPYC		DS	ATVMPSGNRELL
ESM1		CS	EHDMASGDGNIV
GPC1	1	HS	ASDDGSGSGSGD
GPC1	2	HS	DDGSGSGSGDGC
GPC1	3	HS	GSGSGSGDGCLD
GPC2	1	HS	PPALISGEHLRV
GPC2	2	HS	GLVEDSGSFLVH
GPC2	3	HS	DFYGESGEGLDD
GPC2	4	HS	ADEDASGSGGGQ
GPC2	5	HS	EDASGSGGGQQY
GPC3	1	HS	EEGFESGDCGDD
GPC3	2	HS	ECIGGSGDGMIK
GPC4	1	MX	ISDESSGEGSGS
GPC4	2	MX	SSGEGSGSGCEY
GPC4	3	MX	GEGSGSGCEYQQ
GPC5	1	MX	GIKAQSGNPEVK
GPC5	2	MX	LLQLGSGGGMVE
GPC5	3	MX	MVEQVSGDCDDE
GPC5	4	MX	DGCGGSGSGEVK
GPC5	5	MX	CGGSGSGEVKRT
HSPG2	1	HS	LADSISGDDLGS
HSPG2	2	HS	GDDLGSGDLGSG
HSPG2	3	HS	SGDLGSGDFQMV
HSPG2	4	CS	VCRAASGPGPEQ
HSPG2	5	CS	TTPSLSGAGSYL
HSPG2	6	CS	RCQQGSGHGIAE
MIC23		CS	VFAQVSGERLYD
MUC24		MX	KTVTTSGTTNNT
NPLN1		MX	QANCHSGTGDDF
PRG		CS	EEEWGSGSEDAS
PTPZ	1	CS	GAEDSSGSSPAT
PTPZ	2	CS	EDSTSSGSEESL
PTPZ	3	CS	GDGEWSGASSDS
PTPZ	4	CS	TSDEESGSGQGT
PTPZ	5	CS	DEESGSGQGTSD
SDC1	1	CS	EDQDGSGDDSDN
SDC1	2	HS	DSDNFSGSGAGA
SDC1	3	HS	DNFSGSGAGALQ
SDC1	4	CS	PAAEGSGEQDFT
SDC1	5	CS	FTFETSGENTAV
SDC2	1	HS	SIEEASGVYPID
SDC2	2	HS	DYASASGSGADE
SDC2	3	HS	ASASGSGADEDV
SDC3	1	MX	LDDLYSGSGSGY
SDC3	2	MX	DLYSGSGSGYFE
SDC3	3	MX	YSGSGSGYFEQE
SDC3	4	MX	YFEQESGIETAM
SDC3	5	MX	VSGGPSGDFELP
SDC3	6	MX	DNAIDSGSSAAQ
SDC4	1	HS	EGRYFSGALPDD
SDC4	2	HS	DDFELSGSGDLD
SDC4	3	HS	FELSGSGDLDDL
TCN1	1	MX	EEQETSGDFGSG
TCN1	2	MX	SGDFGSGGSVVL
TCN2	1	MX	DIVGFSGDFGSG
TCN2	2	MX	SGDFGSGVGWED
TCN3	1	MX	IDFEISGDFASG
TCN3	2	MX	SGDFASGDFHEW
TGFR3	1	MX	ALGDSSGWPDGY
TGFR3	2	MX	YEDLESGDNGFP
TG		CS	ELTAGSGLREDL
THBD	1	MX	VDGGDSGSGEPP
THBD	2	MX	GGDSGSGEPPPS

Figure S3–Related to Figure 5. Alignment of validated GAG attachment sites in human PGs. The sequences were obtained from UniProt. The two sites used in this study are underlined (AMBP, bikunin; SDC1, syndecan 1). The identity of the GAG is abbreviated as follows: HS, heparan sulphate; CS, chondroitin sulphate; DS, dermatan sulphate; MX, mixed/ambiguous.
